# Metabolite Profiling of Italian Tomato Landraces with Different Fruit Types

**DOI:** 10.3389/fpls.2016.00664

**Published:** 2016-05-19

**Authors:** Svetlana Baldina, Maurizio E. Picarella, Antonio D. Troise, Anna Pucci, Valentino Ruggieri, Rosalia Ferracane, Amalia Barone, Vincenzo Fogliano, Andrea Mazzucato

**Affiliations:** ^1^Department of Agricultural and Forestry Sciences, University of TusciaViterbo, Italy; ^2^Food Quality Design Group, Wageningen UniversityWageningen, Netherlands; ^3^Department of Agricultural Sciences, University of Naples “Federico II”Napoli, Italy

**Keywords:** Amadori products, amino acids, glycoalkaloids, landraces, metabolites, phenolics, quality, tomato

## Abstract

Increased interest toward traditional tomato varieties is fueled by the need to rescue desirable organoleptic traits and to improve the quality of fresh and processed tomatoes in the market. In addition, the phenotypic and genetic variation preserved in tomato landraces represents a means to understand the genetic basis of traits related to health and organoleptic aspects and improve them in modern varieties. To establish a framework for this approach, we studied the content of several metabolites in a panel of Italian tomato landraces categorized into three broad fruit type classes (flattened/ribbed, pear/oxheart, round/elongate). Three modern hybrids, corresponding to the three fruit shape typologies, were included as reference. Red ripe fruits were morphologically characterized and biochemically analyzed for their content in glycoalkaloids, phenols, amino acids, and Amadori products. The round/elongate types showed a higher content in glycoalkaloids, whereas flattened types had higher levels of phenolic compounds. Flattened tomatoes were also rich in total amino acids and in particular in glutamic acid. Multivariate analysis of amino acid content clearly separated the three classes of fruit types. Making allowance of the very low number of genotypes, phenotype-marker relationships were analyzed after retrieving single nucleotide polymorphisms (SNPs) among the landraces available in the literature. Sixty-six markers were significantly associated with the studied traits. The positions of several of these SNPs showed correspondence with already described genomic regions and QTLs supporting the reliability of the association. Overall the data indicated that significant changes in quality-related metabolites occur depending on the genetic background in traditional tomato germplasm, frequently according to specific fruit shape categories. Such a variability is suitable to harness association mapping for metabolic quality traits using this germplasm as an experimental population, paving the way for investigating their genetic/molecular basis, and facilitating breeding for quality-related compounds in tomato fruits.

## Introduction

Over the past half century nutrient content and flavor of intensively bred crops has dropped because breeding efforts focused mainly on yield, stress resistance and agronomic, and technological properties of the edible product. Tomato (*Solanum lycopersicum* L.) is a good example of this trend: yield has remarkably increased but its taste has worsened according to consumers (Zanor et al., 2009; Causse et al., 2010; Tieman et al., 2012; Klee and Tieman, 2013). Compared to traditional varieties, modern cultivars are thought to have fewer of the most important contributors to flavor (sugars, acids, free amino acids, and volatiles).

The cultivated tomato is a model for the study of fruit development and a major crop being the second most cultivated and consumed vegetable worldwide. Domesticated in Tropical America, tomato was introduced in the Old World at the beginning of the Sixteenth-century. Only one century later the species began to be appreciated for its edible product and its cultivation spread through Europe, with greater success in the Mediterranean countries, including Spain, and Italy (Soressi, 1969; Esquinas-Alcazar and Nuez, 1995; Andreakis et al., 2004; García-Martínez et al., 2013). Due to its success in cultivation and to the wide environmental variability, tomato found in Italy a secondary center of diversification and several landraces developed in different regions according to human selection and adaptation to local climatic and edaphic conditions (Siviero, 2001; Mazzucato et al., 2008). This led to the establishment of landraces with different typologies of the fruit, including flat angled and ribbed tomatoes as well as pear-shaped, heart-shaped, extremely elongated, and cherry and plum forms. All these landraces have been cultivated for centuries and are still common in the local markets (Soressi, 1969; Acciarri et al., 2007). Flattened-ribbed tomatoes were mainly diffused in Northern (“Costoluto Genovese”, “Riccio di Parma”, “Ladino di Pannocchia”) and Central (“Costoluto fiorentino”, “Pantano romanesco”, “Scatolone di Bolsena”, “Spagnoletta di Gaeta e Formia”) Italy. Differently, varieties with elongate (“San Marzano”, “Corbarino”), or oval/round (“Piennolo”, “Pizzutello”) fruit shape were mainly found in the Southern regions of the country (Soressi, 1969; Andreakis et al., 2004). Whereas few of these varieties are found in the official registers of varieties, many of them are only listed in voluntary regional catalogs and in registers for conservation varieties.

Although these traditional types usually lack good agronomic performances in terms of yield, resistance and shelf-life of the product, they usually show good adaptation to local environments and outstanding organoleptic qualities. Therefore, it is thought that traditional varieties represent a vault of genes with great interest for improving health- and flavor-related compounds in tomato (Rodríguez-Burruezo et al., 2005; Tieman et al., 2012; Figàs et al., 2015a,b). A strategy to valorize this genetic treasure is to unravel the extent of genetic variability for primary and secondary metabolites in traditional tomato germplasm and to establish correlations between the composition of the fruit, its genetic basis, and the consumer preferences (Hurtado et al., 2014).

Including in a broad sense health and flavor aspects, tomato quality is mainly determined by morphological traits (size, shape, absence of defects) and by the content in products of the primary (sugars, acids, free amino acids) and of the secondary (carotenoids, flavonoids, volatiles) metabolism. Several studies have addressed the identification of genetic factors (quantitative trait loci, QTLs) underlying important traits related to quality in tomato, including morphology, and proximate traits (Shirasawa et al., 2013; Ruggieri et al., 2014; Sacco et al., 2015). Other studies addressed the identification of QTLs related to metabolic traits (mQTLs) with a focus on primary metabolism (Saliba-Colombani et al., 2001; Causse et al., 2002, 2004; Fulton et al., 2002; Schauer et al., 2008; Xu et al., 2013). Among secondary metabolites, most attention has been payed to carotenoids (Rousseaux et al., 2005; Panthee et al., 2013) and volatiles (Mathieu et al., 2009; Zanor et al., 2009; Tieman et al., 2012; Zhang et al., 2015). Fewer studies have addressed the variation in amino acids, among primary (Schauer et al., 2006, 2008; Sauvage et al., 2014), and in alkaloids and phenolic compounds among secondary metabolites (Rousseaux et al., 2005; Alseekh et al., 2015). In addition, no specific analysis has been carried out to search for mQTL associated with Amadori products, a class of compounds formed by the interaction between reducing sugars and amino acids or proteins, that increase with ripening due to the high concentration of sugars, free amino groups, and the acidic environment (Meitinger et al., 2014; Troise et al., 2015).

Due to the wide variability for chemical composition traits described in traditional tomato germplasm (Martínez-Valverde et al., 2002; Rodríguez-Burruezo et al., 2005; Carli et al., 2011; Tieman et al., 2012; Panthee et al., 2013; Cortés-Olmos et al., 2014; Figàs et al., 2015b), the adoption of collections of landraces as experimental populations has been regarded as a promising strategy to associate genetic regions to phenotypic traits of interest (Mazzucato et al., 2008; Panthee et al., 2013; Ruggieri et al., 2014; Sacco et al., 2015). To investigate the potentialities of Italian traditional varieties in association studies involving quality-related compounds, we set up to study the content of several metabolites in a panel of landraces representing three broad fruit typology classes (flattened/ribbed, pear/oxheart, and round/elongate). This characterization paves the way for investigating the genetic/molecular basis for such a variation and for breeding tomatoes with improved fruit quality.

## Materials and methods

### Plant materials

**Figure 1:**
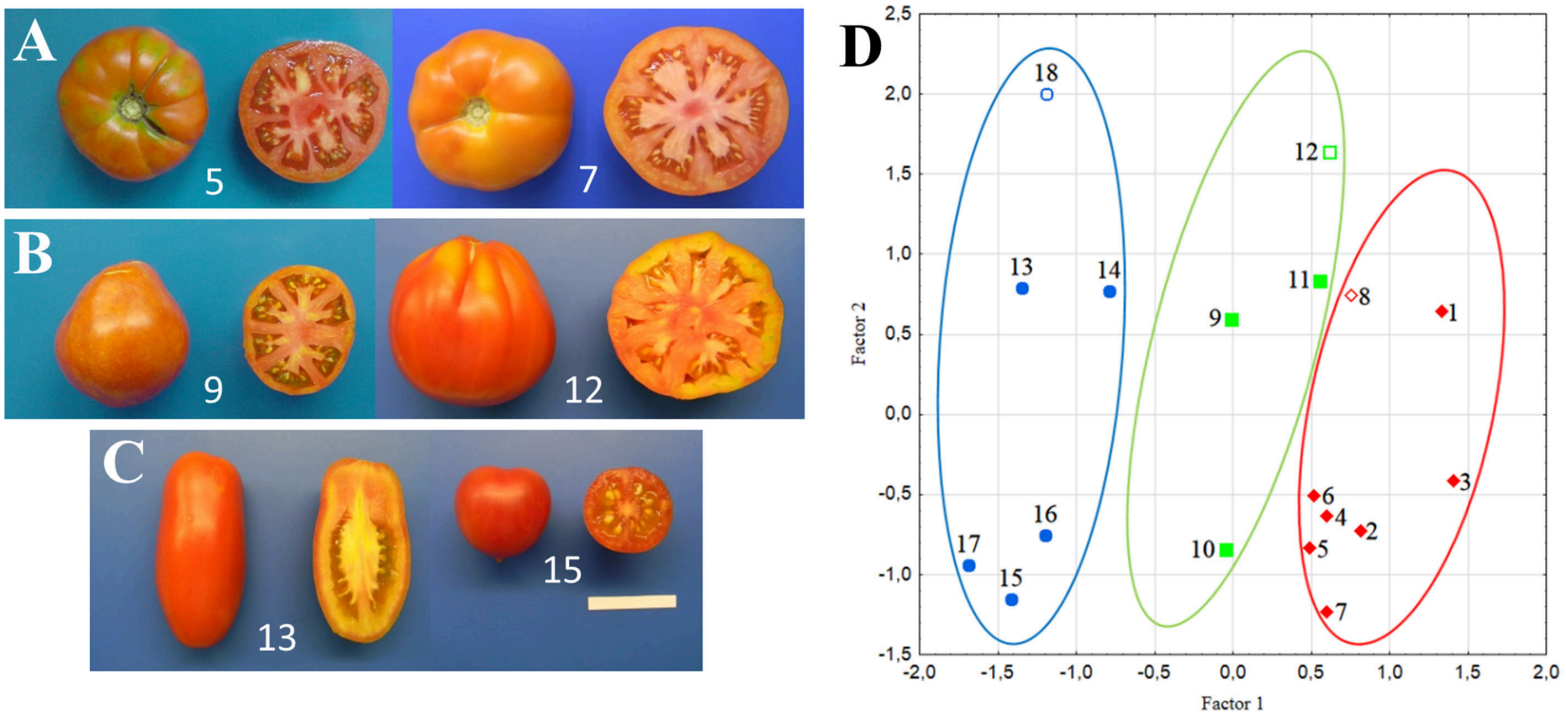
**Examples of tomato genotypes studied and their distribution according to multivariate Factor Analysis**. Flattened/ribbed **(A)**, pear/oxheart **(B)**, and round/elongate **(C)** fruits produced by six of the varieties included in the experiments. Separation of the 18 studied varieties according to the first two factors based on morphological traits **(D)** Circles group accession with flattened/ribbed (red), pear/oxheart (green) and round/elongate (blue) fruits; open symbols refer to hybrids. In all panels, numbers refer to the accession codes given in Table 1.

Fourteen Italian and one French tomato landraces and three modern F_1_ hybrids were adopted for this study (Table 1). Seven landraces belonged to the category of tomatoes with flattened/ribbed fruits, three to pear/oxheart (globose) types, and five to the round/elongate category (Figures 1A–C). Three modern hybrids corresponding to the flat (Marinda, Nunhems), pear (Tomawak, Syngenta), and elongated (Pozzano, Enza Zaden) fruit category were chosen for comparison and purchased from the market. Seeds of landraces were obtained from the tomato collection held by the authors at the University of Tuscia. A field trial was established with the above-described seed stocks at Viterbo, Italy (42°25′07″ N, 12°06′34″ E). The accessions were arranged in a randomized block design with two replicates and eight plants per elementary experimental unit. Plants were grown in open field with the standard agronomic practices adopted for genotypes with indeterminate growth. F_2_ progenies (*n* = 18) of the hybrids included in the study were grown to maturity in the subsequent season to check for the eventual segregation of alleles conferring delayed ripening.

**Table 1:** **Landraces (L) and hybrids (H) used in the analyses, their origin, classification into fruit shape classes, and group means for selected phenotypic traits**.

**Code**	**Name**	**Varietal type**	**Region/Company of origin**	**Fruit shape class**	**Phenotypic traits^c^**		
					**Pericarp index**	**Dry weight (%)**	**Brix value**
1	Mezzo tempo	L	Abruzzo	Flattened/ribbed	0.74 b	4.76 b	4.31 b
2	Spagnoletta	L	Latium				
3	Stella	L	Tuscany				
4	Costoluto fiorentino	L	Tuscany				
5	Scatolone di Bolsena	L	Latium				
6	Pantano romanesco	L	Latium				
7	Marmande	L	France				
8	Marinda	H	Nunhems				
9	Cuor di bue di Albenga	L	Ligury	Pear/oxheart	0.81 b	5.02 b	4.56 ab
10	Cuor di bue	L	Italy^b^				
11	Pera d'Abruzzo	L	Abruzzo				
12	Tomawak	H	Syngenta				
13	San Marzano	L	Campania	Round/elongate	1.18 a	6.09 a	5.20 a
14	Allungato^a^	L	Umbria				
15	Principe Borghese	L	Campania				
16	Ovale Puglia	L	Puglia				
17	Ovale Campania	L	Campania				
18	Pozzano	H	Enza Zaden				

a *Analyzed as belonging to the “Pear/oxheart” group after genotypic analysis*.

b *Landrace diffused in several regions*.

c *Means within a column followed by the same lowercase letter are not significantly different for P ≤ 0.05*.

### Morphological characterization

On a single plant basis, 15 morpho-physiological traits were scored or calculated as detailed in Table S1. Briefly, the growth habit (GH), plant height (PH), inflorescence type (IT), and green shoulder (GS) were scored during plant growth. At the maturity of the second truss, four representative fruits per plant were used to measure or score fruit polar (PD, mm) and equatorial (ED, mm) diameter, stem-end shape (SES, score), blossom-end shape (BES, score), number of fruit locules (LN), pericarp thickness (PT, mm), puffiness (PUF, score), fruit weight (FWE, g) and fruit-shape cross section (FSC, score). Two further traits were calculated; the fruit-shape index [FS, (PD/PE)] and the pericarp thickness index [PI, (PT/((PD + PE)/2))]. These descriptors largely conform to the guidelines of Bioversity International for tomato (http://www.bioversityinternational.org/e-library/publications/detail/descriptors-for-tomato-lycopersicon-spp/).

Six fruits per genotype were cut and the soluble solids content was measured as refractive index at 20°C (Brix) in the juice obtained after extracting the seeds using a digital refractometer (MA871, Milwaukee, Milwaukee Instruments, Inc., NC, USA) on a single fruit basis. Dry matter content was calculated as the percentage of dry weight (DW) over fresh weight (FW). Total solid content determination was carried out by gravimetric method according to AOAC International (1995).

### Chemicals

Acetonitrile and water for liquid chromatography high resolution mass spectrometry (LC/HRMS) analysis were obtained from Merck (Darmstadt, Germany). L-Amino acids standards, perfluoropentanoic acid (NFPA), acetic acid, and formic acid were obtained from Sigma-Aldrich (St. Louis, MO). Amadori products (APs) were synthesized according to the procedure described in Troise et al. (2015). The calibration solutions (see “Liquid chromatography/high resolution mass spectrometry” Section) were obtained from Thermo Fisher Scientific (Bremen, Germany).

### Genotypic data retrieval

Genotypic data of the landraces adopted here were available from the study of a wider collection of tomato germplasm using the SolCAP single nucleotide polymorphism (SNP) array (Sacco et al., 2015). Raw data were retrieved and markers with more than 10% missing genotypes were removed. After discarding sites with Minor Allele Frequency (MAF)<15%, levels of observed heterozygosity (H_O_) were calculated and a neighbor-joining tree was generated using TASSEL 5.0 (Bradbury et al., 2007).

### Liquid chromatography/high resolution mass spectrometry LC-HRMS analyses

Twenty representative vine-ripened fruits were harvested for all the genotypes from eight plants per accession and the concentration of amino acids and APs, glycoalkaloids, and phenolic acids (63 markers in total) was monitored by liquid chromatography high resolution mass spectrometry (LC-HRMS). Each sample was extracted twice and analyzed in duplicate (*n* = 4). Data were reported as mg/kg FW.

Amino acids and APs were analyzed according to Troise et al. (2015). Briefly, tomato samples were ground in a knife mill Grindomix 200 (Retsch, Haan, Germany) and 100 mg were mixed with 0.3 mL of deionized water and centrifuged (14,800 rpm, 20 min, 4°C). The supernatants were filtered using regenerated cellulose filters (RC 0.45 μm, Phenomenex, Torrance, CA). For the chromatographic separation of amino acids and their respective APs, the mobile phases consisted of 5 mM NFPA (solvent A) and 5 mM NFPA in acetonitrile (solvent B). The following linear gradient of solvent B (min/%B): (0/2), (2/2), (5/50), (7/50), (9/50) was used. The flow rate was set to 200 μL/min and the injection volume was 5 μL. Chromatographic separation of amino acids and APs was achieved through a thermostated (30°C) core-shell C-18 column (Kinetex 2.6 μm, 100 × 2.1 mm, Phenomenex, Torrance, CA). The Accela 1250 UPLC system (Thermo Fisher Scientific, Bremen, Germany) was directly interfaced to an Exactive Orbitrap high resolution mass spectrometer (Thermo Fisher Scientific, Bremen, Germany). Analytes were detected through a heated electrospray interface (HESI) operating in the positive mode and scanning the ions in the *m/z* range of 60–500. The resolving power was set to 50,000 full width at half maximum (FWHM, *m/z* 200) resulting in a scan time of 1 s. The automatic gain control was used in balanced mode (1 × 10^6^ ions); maximum injection time was 50 ms. The interface parameters were as follows: spray voltage 3.8 kV, capillary voltage 10 V, skimmer voltage 15 V, capillary temperature 275°C, heater temperature 200°C, sheath gas flow 30, and auxiliary gas flow 3 arbitrary units.

The same simplified extraction procedure was used for antioxidants compounds. Phenolic acids and glycoalkaloids were analyzed according to Troise et al. (2014). Chromatographic separation was carried out on a Gemini C18 column (5 μm, 150 × 2.0 mm Phenomenex, Torrance, CA) thermostated at 30°C while mobile phases were 0.1% formic acid (solvent A) and 0.1% formic acid in acetonitrile (solvent B). The following linear gradient of solvent B (min/%B): (0/10), (8/90), (10/90) was used. The flow rate was set to 200 μL/min and the injection volume was 10 μL. The UPLC was directly interfaced to the Orbitrap equipped with HESI interface. Mass analyzer operated in the full spectra acquisition mode and positive and negative ionization mode was simultaneously used in the mass range of *m/z* 65–1300. The resolving power was set to 50,000 (FWHM, *m/z* 200) resulting in a scan time of 1 s. The automatic gain control was used (ultimate mass accuracy mode, 5 × 10^5^ ions) and maximum injection time was 100 ms. The interface parameters were as follows: the spray voltage was 3.5 and −3.0 kV in positive and negative ion mode, respectively; the tube lens was at 100 V (−100 V in negative ion), the capillary voltage was 30 V (−50 V in negative ion), the capillary temperature was 275°C, and a sheath and auxiliary gas flow of 30 and 15 arbitrary units were used. The instrument was externally calibrated by infusion with a positive ions solution that consisted of caffeine, Met-Arg-Phe-Ala (MRFA), Ultramark 1621, and acetic acid in a mixture of acetonitrile/methanol/water (2:1:1, v/v/v), then with a negative ions solutions that consisted of sodium dodecyl sulfate, sodium taurocholate, Ultramark 1621, and acetic acid in a mixture methanol/water (1:1 v/v). Reference mass (lock mass) of diisooctyl phthalate ([M + H]^+^, exact mass = 391.28429) was used as recalibrating agent for positive ion detection. To optimize the mass spectrometer conditions and the mass accuracy, the calibration procedure was performed each day both in positive and negative mode. The analytical performances, i.e., mass error, linearity, reproducibility, repeatability, LOD, and LOQ were in line with those previously reported.

### Data analysis

Analysis of variance (ANOVA) for differences among fruit shape groups was carried out adopting the General Linear Model (GLM) using the SAS software (SAS Institute, 2004). Significant differences were estimated by Duncan multiple range test. A Pearson correlation matrix was developed to ascertain the correlation coefficient (*r*) between all studied parameters and a heatmap obtained by Gitools software version 2.2.2 (Perez-Llamas and Lopez-Bigas, 2011). Standardized morphological and metabolic data were statistically analyzed by Factor Analysis (FA) using “Statistica 10” (StatSoft Inc., Tulsa, OK, USA). Hierarchical clustering (Ward's method) of the 15 landraces and the three hybrids under study, based on the content of 63 analyzed metabolites, was carried out by Past 3.11 (Hammer et al., 2001).

To assess the genetic relationships within the investigated collection, the population structure was determined by using STRUCTURE 2.3.4 software (Pritchard et al., 2000), with no a priori information regarding population origin. The degree of admixture was estimated by setting for both burn-in period and Markov Chain Monte Carlo iterations a value of 100,000 for each run. Seven independent runs across a range of *K*-values (*K* = 1–12) were made. The best number of clusters (*K*) was obtained using STRUCTURE HARVESTER program (Earl and vonHoldt, 2012) based on the method of Evanno (Evanno et al., 2005). Genome–Wide Association Study (GWAS) between traits and DNA polymorphisms was performed using the GLM model with Q matrix as implemented in TASSEL 5.0 (Bradbury et al., 2007). *P*-values were corrected following the standard Bonferroni procedure. Significant associations were detected with corrected *p* value lower than 5.2E^−5^ (0.05/954). A physical map of the tomato genome showing the position of the SNP markers significantly associated with the traits was constructed using Map Chart 2.2 (Voorrips, 2002).

## Results

### Phenotyping of morphological traits

All the measured morphological traits, including plant and fruit characters, showed a large range of phenotypic variation among the 15 tomato landraces and three market F_1_ hybrids. With the exception of GH, PH, GS, BES, PT, and PUF, all the phenotypic traits showed significant differences among fruit type groups (Table S2). In addition to obvious differences in traits related to fruit shape (PD, ED, FS), ANOVA indicated that round/elongated types were differentiated for the simple inflorescence, the flat stem end shape, the lower number of locules and lower fruit weight (Table S2). Types with round/elongate fruits also showed higher PI, DW and Brix values (Table 1). The two first FA components explained 56% of the phenotypic variation and distinguished the genotypes according to these phenotypes (Figure 1D). Factor 1 was mainly loaded by FS and LN, whereas Factor 2 mainly by PD.

Several morphological traits were significantly correlated; in addition to trivial correlations (e.g., LN with ED and FWE), plants producing fruits with high LN (flattened/ribbed types) showed also compound IT and depressed SES (Figure S1). DW was highly correlated with Brix. Plant traits (GH and PH) together with GS, PI and PUF were rather independent. In agreement with the previously assumed information, the three hybrids used, when progeny tested in the F_2_ generation, showed no segregation of genes for delayed ripening (not shown).

### Genotyping

SolCAP data for the 15 landraces retrieved from the literature (Sacco et al., 2015) included 7719 readable SNP sites of which 2022 resulted polymorphic in the studied material. Sites filtered for MAF<15% resulted in 954 polymorphic SNPs, that offered a whole coverage of the tomato genome, ranging from a minimum of 54 (Chr6 and Chr10) to a maximum of 155 (Chr3) SNPs per chromosome. All genotypes showed low levels of Ho, ranging between 0.064 and 0.088, with the exception of genotypes #10 and #14 that showed higher values (0.430 and 0.362 respectively, data not show).

**Figure 2:**
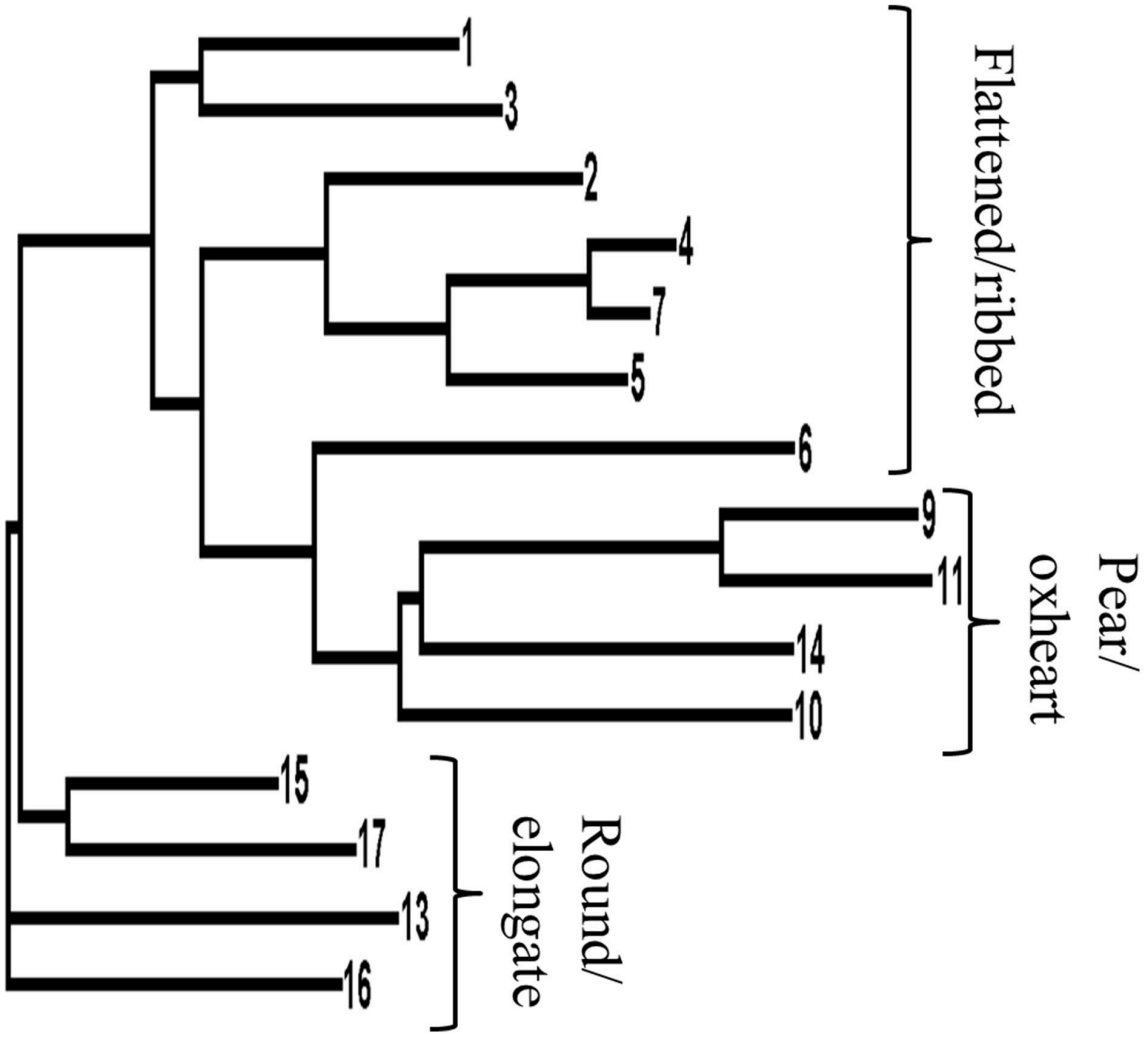
**Neighbor-joining dendrogram based on genetic distances among the 15 landraces for which SNP data were retrieved (Sacco et al., 2015)**. The dendrogram is based on 954 SNP sites polymorphic among the 15 tomato landrace accessions. Numbers indicating each branch refer to the accession code as reported in Table 1.

The Neighbor-joining dendrogram separated genotypes with flat and globose fruits from those with round/elongated berry types (Figure 2). The landrace #14 (Allungato), that was initially classified among the round/elongated types due to the shape index of the fruit (Table 1), clustered among globose types. Therefore, also on the basis of the similar fruit structure (higher number of locules and higher proportion of flesh than in round/elongate types), this genotype was included in the pear/oxheart group in all the further analyses.

### Biochemical analysis

In total, 63 fruit metabolites belonging to the glycoalkaloid, phenolic, free amino acid, and AP classes have been analyzed in the studied genotypes. Detailed data on these analyses are reported in Tables S3–S6.

**Figure 3:**
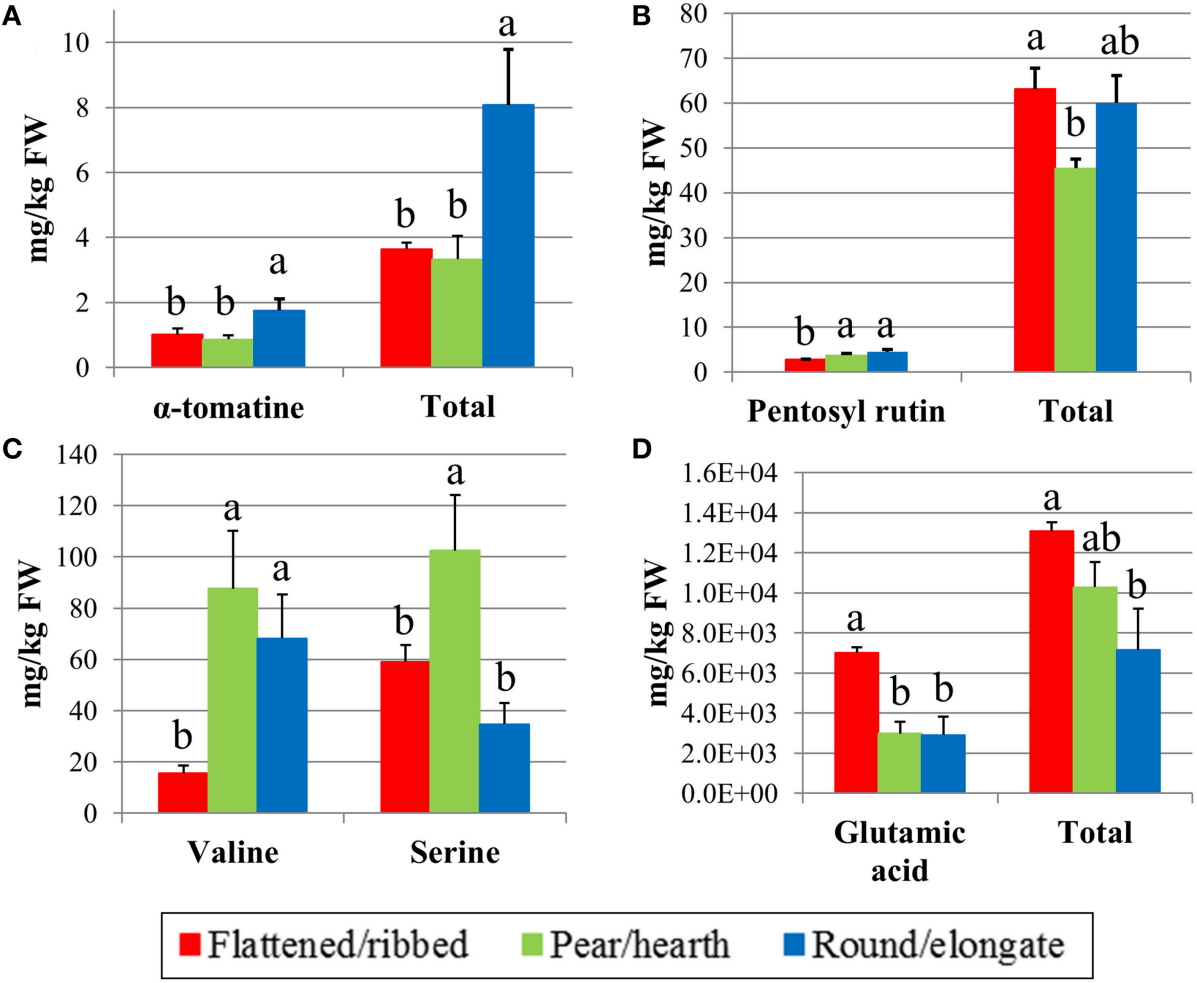
**Significant variation in selected metabolites in the 18 tomato varieties analyzed according to their classification in different fruit typologies**. Content in α-tomatine and total glycoalkaloids **(A)**, pentosyl rutin and total phenolics **(B)**, valine and serine **(C)**, glutamic acid, and total free amino acids **(D)**. Mean values indicated by different letters are significantly different for *P* ≤ 0.01.

With the exception of dehydro-tomatine, all the glycoalkaloids showed significant differences among tomato fruit types (Table S3). The total alkaloid content was very variable among the genotypes; the variety with highest content (#15, Principe Borghese) showed an amount of total glycoalkaloids that was almost eight-fold that recorded in the lowest one (#11, Pera d'Abruzzo). α-tomatine and tomatoside-A were the most represented analytes accounting for more than 50% of total glycoalkaloids. Round/elongate types showed contents significantly higher than the other types both for single analytes and for total alkaloid content (Figure 3A; Table S3). Being higher in tomato plants with round/elongate fruits, glycoalkaloid content was positively correlated with FS, PI, DW and Brix (Figure S1), which are all traits with higher values in round/elongated types. In addition, all the single analytes and the total content showed high reciprocal positive correlations (Figure S1).

Chlorogenic acid together with caffeic acid hexoside were on average the most represented phenolic compounds in tomato ripe fruits, accounting for about 55% of the total average content (Table S4). Variation in total phenolics was lower than that for glycoalkaloids, the highest value being barely two-folds the lowest one. The analyte with highest genotypic variation was Naringin that in the flat type #3 (Stella Pisa) had levels about 34-fold higher than in the pear-shaped genotype #9 (Cuor di bue di Albenga). There were no differences among groups of varieties for the phenolic compounds, with the exception of pentosyl rutin, that showed lower levels in flat tomatoes and total phenolics that were lower in pear/oxheart cultivars (Figure 3B; Table S4).

The most represented amino acids in tomato fruits resulted glutamic acid (Glu) and glutamine (Gln), accounting for up to 70% of the total amino acid content. The accession means showed a wide variation for amino acid composition and several fold differences were observed between the minimum and maximum value. The highest differences were observed for valine (Val), thyrosine (Tyr), and arginine (Arg; Table S5). ANOVA showed significant differences among typologies for 11 amino acids (Table S5), but highly significant (*P* ≤ 0.01) variations were only recorded for Val, serine (Ser), Glu, and total amino acid content (Figures 3C,D). Glu was on average more than two- fold higher in flattened/ribbed varieties than in the other groups of genotypes (Figure 3D).

All amino acids, with the exception of Val, phenyalanine (Phe), Tyr, methionine (Met) and proline (Pro), were strongly positively correlated among them. The content of (at least) 12 amino acids was positively correlated with FWE and negatively correlated with Brix. Glu content was significantly correlated with eight out of 15 morphological variables (Figure S1), indicating that this analyte is strictly related to specific plant and fruit types.

**Figure 4:**
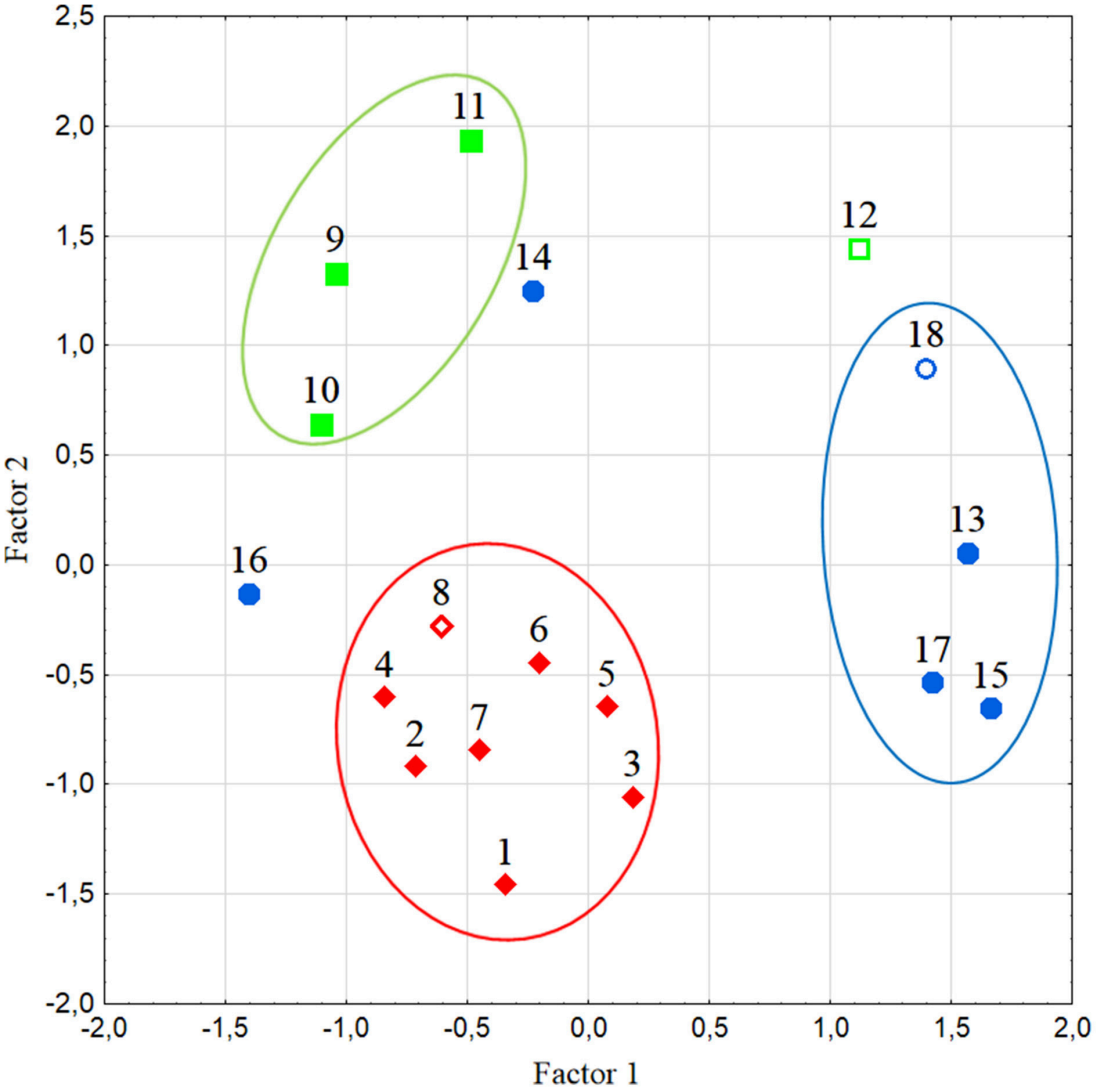
**Distribution of the studied tomato varieties according to the first two factors in multivariate analysis of the amino acid content**. Numbers refer to the accession codes given in Table 1. Circles group accessions with flattened/ribbed (red), pear/oxheart (green), and round/elongate (blue) fruits. Open symbols refer to hybrids.

Multivariate analysis of amino acid content yielded the two first components that explained 72.8% of the total variation. Metabolite contribution to Factor 1 was high and negative for leucine (Leu), isoleucine (Ile), threonine (Thr), asparagine (Asn), Gln, and histidine (His); Factor 2 was positively charged by Phe and negatively by Glu (not shown). The analysis revealed that amino acids clearly separated the three fruit types; the flattened/ribbed types grouped together, whereas the other types were also differentiated with only few exceptions (Figure 4). As supported by genotypic analysis, the elongate type #14 was more related to pear/oxheart shaped tomatoes than to elongate types. On the contrary, the pear-shaped hybrid (#12) was rather distant from landraces of the same typology according to amino acid content (Figure 4). Genotype #16 (Ovale Puglia) also showed in the plot a position distant from the group of varieties with similar fruit type, due to its very high amino acid content (Table S5).

The difference from the highest and the lowest value for total APs was about five-fold; considering single analytes the highest variations were found for Fru-Arg, Fru-Lys and Fru-Gly (Table S6). Fru-Ser was by far the most relevant glycosylated amino acid form, accounting on average for about 75% of the total in mature fruits. Differently from the free amino acids, APs showed less variation among the tomato types analyzed; highly significant differences were only reported for Fru-Leu and Fru-Ile (highest in round/elongate types) and for Fru-Asn (higher in flattened types; Table S6). As for free amino acids, several APs showed a positive correlation with FWE and related morphological traits (Figure S1).

Hierarchical clustering based on 63 metabolites showed that fruit composition is similar in genotypes having similar fruit type (Figure S2). However, hybrids did not always followed this behavior. Whereas the hybrid with elongate fruit (#18, Pozzano) grouped within landraces with the same fruit type, the hybrids representing oxheart and flattened types were misplaced and did not show a metabolic composition parallel to that shown by landraces with similar fruits (Figure S2).

### Molecular analysis and comparison with morphological and biochemical traits

Making allowance for the very small number of genotypes sampled, we crossed morphological and biochemical data with molecular polymorphisms. To improve the reliability of such attempt, the structure of the population has been taken into account and the Bonferroni correction applied to a level of significance below *P* < 5.2E-05. The Evanno test (Evanno et al., 2005) indicated that the best number of clusters to divide the population was three (Figure S3A), in parallel with the a priori division on fruit typologies. Model-based groups represented in the plot of ancestry estimates (Figure S3B) confirmed the genetic relatedness of types with round/elongate fruits, with the exception of accession #14 that was more similar to pear/oxheart types. Among flat-fruited tomatoes, #6 (Pantano Romanesco) also showed relatedness to pear/oxheart types, as already indicated by hierarchical clustering (Figure 2).

GWAS yielded a total of 66 markers (involving 56 genes) significantly associated with the morphological traits and the four categories of analytes on 11 tomato chromosomes (Table 2). No association was reported on Chr9. A relatively low number of associations was highlighted for each category of traits analyzed; the position in the tomato genome of the markers significantly associated with morphological and metabolic traits is mapped in Figure S4.

**Table 2:** **SNP Markers associated to morphological and biochemical traits in Italian tomato landraces**.

**Phenotypic class**	**Trait^a^**	**SolCap ID^b^**	**Chr^c^**	**Position**	**Solyc ID**	***p*-value**
Morphological traits	ED	5624	02	47148187	Solyc02g083900.2.1	2.82E-05
		5625	02	47218361	Solyc02g083990.2.1	5.07E-05
		4597	02	52417091	Solyc02g090960.1.1	4.47E-06
		7584	03	64512996	Solyc03g114560.2.1	5.31E-06
		2501(2)	04	1170841	Solyc04g007500.1.1	2.82E-05
		2020	06	41163560	Solyc06g065720.1.1	2.82E-05
		6943	07	62062707	–^d^	3.77E-05
		112	12	66795414	Solyc12g099800.1.1	2.82E-05
	GS	1722	07	64287991	Solyc07g056430.2.1	4.36E-05
	PI	7583	03	64588871	Solyc03g114690.2.1	2.12E-05
	LN	518	01	730154	Solyc01g006050.2.1	1.40E-06
		2557	02	40887642	Solyc02g071440.2.1	9.13E-07
		5731	02	45515428	Solyc02g081640.2.1	3.71E-07
		2994	02	45761358	Solyc02g082030.2.1	3.71E-07
		7465	03	62386150	Solyc03g111740.2.1	1.45E-06
		6980	04	1163761	Solyc04g007490.2.1	3.71E-07
		5963	07	57927159	Solyc07g044870.2.1	2.51E-07
		1981	10	58672684	Solyc10g074950.1.1	8.30E-08
		3617	11	54854070	Solyc11g071340.1.1	1.45E-06
		2076 (2)	11	54970033	Solyc11g071530.1.1	1.45E-06
		3534	11	55072385	Solyc11g071660.1.1	9.13E-07
		504	11	55074586	Solyc11g071670.1.1	1.45E-06
Glycoalkaloids	Total	377	08	230545	Solyc08g005300.1.1	3.80E-05
		1745	10	51335068	–	3.80E-05
		7469	10	51524389	Solyc10g051110.1.1	3.80E-05
		5993 (3)	10	54449604	Solyc10g054010.1.1	3.80E-05
		5984 (2)	10	54518281	Solyc10g054030.1.1	3.80E-05
		1982	10	58189616	–	3.80E-05
Phenolics	Coumaric acid hexoside	1678	03	52079075	Solyc03g080190.2.1	4.68E-06
		7575	03	65365147	–	5.20E-07
		2374	03	66796966	Solyc03g117760.2.1	5.68E-06
		2373	03	66806264	Solyc03g117770.2.1	5.18E-05
		2372 (2)	03	66807096	Solyc03g117780.2.1	5.18E-05
		4652	03	70262451	Solyc03g123390.2.1	6.96E-06
		4651	03	70365262	Solyc03g123530.2.1	7.32E-06
		2193	10	57397425	Solyc10g055760.1.1	5.68E-06
		7535	10	64340240	Solyc10g084990.1.1	5.68E-06
		7534	10	64340314	–	5.68E-06
	Naringin	2099	04	7569869	Solyc04g017610.2.1	6.35E-06
		6122	04	36996123	Solyc04g047750.2.1	6.35E-06
		1875	08	63328385	Solyc08g079900.1.1	6.35E-06
Amino acids	Ala	7498	02	39605044	Solyc02g069780.2.1	3.51E-05
		7499 (2)	02	39617885	Solyc02g069780.2.1	3.51E-05
		6407	03	54696154	Solyc03g093400.2.1	1.45E-05
		1572	05	3898119	Solyc05g009700.2.1	2.71E-05
		2915	05	3947680	Solyc05g009740.1.1	2.71E-05
		1327	06	37080820	Solyc06g054270.2.1	4.73E-05
		7664	10	4260136	Solyc10g011960.1.1	8.55E-06
		7122	11	12993630	–	3.41E-05
	Asn	1106	11	55733112	–	1.60E-05
	Glu	3340	10	6769606	Solyc10g018140.1.1	5.20E-05
		531	10	7464111	Solyc10g018340.1.1	5.20E-05
		1983	10	58003053	Solyc10g074470.1.1	5.20E-05
		3455	10	58307818	Solyc10g074700.1.1	5.20E-05
	Pro	1918 (4)	07	2870461	Solyc07g008160.2.1	3.30E-05
		1914	07	2883786	Solyc07g008170.2.1	3.30E-05

*For each marker the position in bp on the related chromosome is reported, together with the corresponding gene (Solyc ID) according to SL2.50 and the p-value*.

a *Abbreviation as detailed in Materials and Methods*.

b *Numbers in brackets indicate multiple significant markers within the same gene*.

c *Chromosome*.

d *-Not in gene region*.

Among morphological traits, high numbers of associations were reported for ED (9) and LN (13). For glycoalkaloids, only the total content of showed positive associations, indicating two regions of the genome, one on the short arm of Chr8 and the second on the long arm of Chr10 (Table 2). Two phenolic compounds, coumaric acid hexoside and naringin, showed associations; remarkably, coumaric acid hexoside had eight associated markers spanning a wide region of the long arm of Chr3. Four amino acids yielded significant hits, alanine (Ala) on six different chromosomes and Asn, Glu and Pro, each one on a single chromosome. For Glu, significant markers were found on both the short and long arm of Chr10 (Table 2; Figure S4).

## Discussion

A deep characterization of tomato germplasm used in traditional cultivations, including morphological, agronomic, nutritional, and organoleptic traits, is desirable for several reasons. This phenotypic information, coupled with deep genotypic analysis, can be helpful to characterize and distinguish landraces for their quality related traits in fresh (Mazzucato et al., 2010; Figàs et al., 2015a and refs therein) and processed (Andreakis et al., 2004; Caramante et al., 2011) products, to improve the traditional varieties without losing those peculiar traits (Acciarri et al., 2007) and for breeding quality improvement alleles into more productive and modern backgrounds (Rodríguez-Burruezo et al., 2005; Tieman et al., 2012; Sacco et al., 2015). Finally, landrace germplasm can be adopted to discover structural and regulatory genes important in tuning plant primary and secondary metabolism, as suitable targets for metabolic engineering strategies (Bovy et al., 2007).

In this work, we pursued the analysis of a set of Italian tomato landraces representing the major fruit typologies in order to describe the degree of variation in metabolite concentration in comparison with modern hybrids belonging to the same fruit shape classes. Selected hybrids were confirmed to lack genes affecting ripening [such as *ripening inhibitor* (*rin*) and *non-ripening* (*nor*)] which could have influenced the metabolic composition of red ripe fruits (Osorio et al., 2011). Our analysis evidenced the wide variation of several metabolites in different genotypes and overall in groups with different fruit types, in agreement with description of large diversity in tomato germplasm autochthonous of different geographic regions (Rodríguez-Burruezo et al., 2005; Cortés-Olmos et al., 2014; Figàs et al., 2015b).

### Content of quality-related metabolites in tomatoes with different fruit shape

A wide variation among varieties and types was found for glycoalkaloid compounds, the round/elongate varieties having up to eight-fold the content showed by other genotypes, in agreement with previous estimations on cherry and elongate tomatoes (Leonardi et al., 2000). However, the content of alkaloids found in Italian landraces belonging to this category are higher than those reported in the literature (Friedman, 2002). As round/elongate varieties also show higher values of Brix and DW, these two correlated traits (Carli et al., 2009, 2011; Figàs et al., 2015b; this work) showed a strong correlation with all the alkaloid analytes. Although alkaloids are regarded as potentially toxic compounds, many health-beneficial effects of tomatine have also been described. In addition, the content in alkaloids may affect the degree of resistance to pathogens and parasites, and the alkaloid-correlated traits Brix and DW are positively correlated with fruit taste (Figàs et al., 2015b). Thus, selecting new tomato varieties with beneficial total glycoalkaloid content could be an important breeding objective in the future.

The tomato fruit contains also a considerable amount of phenolic compounds, among which chlorogenic acid and quercetin are the most represented (Martínez-Valverde et al., 2002). It was reported that phenolics give the major contribution to antioxidant capacity (Toor and Savage, 2005). In our analysis, flattened types showed a concentration of total phenolics higher than pear/oxheart types, whereas round/elongate tomatoes were intermediate. Because a taste index showed positive correlation with total phenolics (Figàs et al., 2015b), the improvement in this class of compounds will also be important to breed tomatoes with improved both nutritional and organoleptic quality (Kaushik et al., 2015).

Free amino acids form about 2–2.5% of the total dry matter of tomatoes. In addition to represent a source of nitrogen in the diet, amino acids play a role in organoleptic qualities deeply affecting fruit flavor (Choi et al., 2014). The content of several amino acids showed a strong positive reciprocal correlation in the material analyzed, confirming that these metabolites share high interconnection (Schauer et al., 2006; Carli et al., 2009). The most abundant amino acid found in the tomato fruits analyzed was Glu, followed by Gln; these two forms comprised on average 70% of the total free amino acids confirming previous reports (Kader et al., 1978; Sorrequieta et al., 2010; Pratta et al., 2011; Choi et al., 2014). High variation in Glu content among cultivars with different fruit size was also reported in the literature (Zushi and Matsuzoe, 2011). Glu, commonly referred to as “glutamate” because it is present in its anionic form at physiological pH, plays diverse biological roles in organisms (Forde and Lea, 2007). In fruits, it represents a taste-enhancing compound, known to be sensed as the fifth basic taste (umami), which evokes a savory feeling; this property has been related to an adaptive role in attracting mammal predators (Chaudhari et al., 2009). Average content in Glu in tomato fruits found in literature ranges between 1000 and 2000 mg/kg FW (Kader et al., 1978; Pratta et al., 2011; Zushi and Matsuzoe, 2011), reaching a maximum of 3500 in a cherry green-fruited variety (Choi et al., 2014). The average concentration of Glu detected in Italian flattened/ribbed genotypes (6871 mg/kg FW), as well as that in the French cultivar Marmande (7565 mg/kg FW), are the highest ever reported being about two-fold those measured in other tomato types.

Amadori compounds increase in the tomato paste during processing due to the Maillard reaction. Due to their processing-induced nature, APs are found in raw fruits at level several folds lower than free amino acids. Despite processing-induced APs in foods have historically been related with mostly negative health effects, a few individual analytes have been associated with antioxidant activity and other positive biological properties. The activity of Fru-His as a potent copper chelator indicated possible antioxidant activity (Mossine and Mawhinney, 2007). If the positive correlation between Fru-His in the fresh fruit and in the processed tomato will be demonstrated, the significant differences in Fru-His detected in the material studied here could be a basis to obtain fortified tomatoes as a consequence of the antioxidant potential of Fru-His and the inhibitory activity of Fru-His/lycopene against prostate cancer cell proliferation (Mossine et al., 2008).

### Molecular analysis and comparison with morphological and biochemical traits

GWAS strategies rely on the development of large volumes of phenotypic and genotypic data, that can be analyzed together to unravel QTLs and candidate genes involved in the control of complex traits of interest. Although only the analysis of large sets of genotypes may indicate reliable associations, the possibility that a limited sampling can be adopted to obtain useful insights into gene-phenotype relationships and networks has been proposed (Carli et al., 2009, 2011). Even if based on a minimal number of genotypes, the trait-marker relationships reported here are considered to represent a reliable indication of functional genomic regions because of their relatively low number and their frequent coincidence with associations previously reported using biparental populations or GWAS with a wider array of genotypes. Such insights represent a useful basis to extend GWAS on biochemical traits using traditional tomato germplasm.

Several associations with morphological phenotypic traits evidenced here corresponded to already characterized genomic regions. For instance, association of the correlated traits LN and ED with markers of Chr2, Chr10, and Chr11 coincided with those reported by others (Shirasawa et al., 2013; Xu et al., 2013; Sacco et al., 2015) being tightly close to the *Locule number* (*Lc*; Solyc02g083940 or 950), *SUN1* (Solyc10g079240), and *FASCIATED* (*FAS*; Solyc11g071819) gene respectively. In this study, seven out of 12 markers linked to LN and three out of eight markers linked to ED corresponded to polymorphisms previously associated with these traits (Sacco et al., 2015). In addition, the marker associated with Brix with higher probability, that was not described in detail because it did not reach the significance threshold (*P* = 0.0148), was located on Chr10 at position 62.5 Mbp (not shown) in tight proximity to a marker associated with the same trait at position 60.3 Mbp (Xu et al., 2013).

Of the six markers associated with total glycoalkaloids, five mapped on a 7 Mbp region on the long arm of Chr10. This region well-corresponded to that involved in the introgression lines IL10-2 and IL10-3 (Eshed and Zamir, 1995) where QTLs for the content of lycoperoside G and F or esculeoside A were positioned (Alseekh et al., 2015). A gene candidate to underlie these QTLs has been identified in an uncharacterized UDP-glycosyltransferase involved in glycoalkaloids biosynthesis (Solyc10g085230; Itkin et al., 2013; Alseekh et al., 2015). This gene, whose product catalyzes the conversion of esculeoside A to esculeoside A+exose, is compatible with the distalmost QTL position found in our analysis.

Ten markers linked to coumaric acid hexoside were detected on the long arm of Chr3 and Chr10. A QTL involved in coumaric acid-exoside compatible with this latter position was recently described and genes candidate have been proposed as five UDP-glycosyltransferase 1 family genes (*UGT1*; Solyc10g085730, Solyc10g085860, Solyc10g085870, Solyc10g085880, and Solyc10g086240) and one phenylalanine ammonia lyase gene (*PAL*; Solyc10g086180; Alseekh et al., 2015). These genes span positions from 64.81 to 65.10 Mbp, whereas our closest marker mapped at 64.34 Mbp. Three markers linked to the content of naringin were found on Chr4 and Chr8; this represents the first report of markers linked to this metabolite and their consistence will need further investigation.

Out of 15 markers linked to amino acid content, eight showed association with Ala; six of them indicated positions on Chr2, Chr3, Chr5, and Chr10 compatible with previously reported QTLs (Schauer et al., 2006). The same held for the markers linked to Pro content on Chr7. Four markers significantly associated with Glu were arranged on Chr10, two on the short and two on the long arm. The latter position corresponded to a described QTL for Glu content (Fulton et al., 2002). As these markers were remarkably coincident with those linked to total glycoalkaloids, it remains to be ascertained if they actually reflect the position of different genes or are the consequence of the negative correlation existing between total glycoalkaloids and Glu content. However, the markers linked with Glu, spanning a region between Solyc10g074470 and Solyc10g074700 (57.33–57.63 Mbp), were in close proximity to one of the four glutamate dehydrogenase 1 (*GDH1*) genes annotated in tomato (Solyc10g078550, position 59.66 Mbp; Ferraro et al., 2012). *GDH1* encodes an enzyme that converts alpha oxoglutarate to glutamate (Forde and Lea, 2007), an important reaction in glutamate metabolism. Moreover, GDH protein content and activity were highly induced in ripe fruits paralleling the increase in the relative content of Glu at ripening; *GDH1* is thus a good candidate for determining Glu levels in tomato fruits (Sorrequieta et al., 2010).

### Perspectives for improving and valorize Italian tomato landraces

Taking into consideration all the metabolites analyzed, the study indicates that modern hybrids that are selected for particular fruit type categories may not present similar composition and consequently organoleptic qualities as the traditional tomatoes with similar fruit shape (Figure S2). The results also showed that metabolic profiling of tomato landraces can indicate which metabolites contribute more to the quality of specific variety and, once this information will be associated with a sensorial analysis, it will be clear which metabolites contribute more to consumer acceptance.

As an example, the group of flattened/ribbed tomatoes was relatively homogeneous for metabolic composition; however, the Scatolone di Bolsena landrace emerged as having, within this group, the highest Brix value, α-tomatine content and sweetness score according to a non-professional panel test assessment (not shown). This association was in agreement with reports of these traits as positively correlated (Figàs et al., 2015b). The hybrid Marinda, that was misplaced in the hierarchical clustering based on all metabolites, scored lowest values among flat types for all the three traits. Thus it is possible to argue that the fruit composition of this hybrid does not represent that of traditional flat-fruited tomatoes, although Marinda showed other positive properties as high scores for juiciness (not shown).

The content in Glu, a compound directly related to organoleptic quality, was discriminant of genotypes with different fruit types, being high in all flat types and intermediate or low in pear/oxheart and in round/elongate types. One exception was the landrace #16 (Ovale Puglia, a genotype with elongate fruit and high Glu level). Interestingly, at all the four SNP positions linked to Glu content this genotype carried the same allele as the flat tomatoes, giving a good marker and a candidate gene to pursue Glu content improvement. On the contrary, the pear-shaped hybrid Tomawak showed a very low Glu value in comparison with tomatoes with similar fruit types. As it was shown by multivariate analysis of all the analytes, this hybrid showed a different position compared with similar varieties (Figure S2), possibly reflecting different organoleptic qualities. The detection of mQTLs for important metabolites as those exemplified above will give valuable tools to improve traditional tomato varieties by assisted breeding without losing general and specific quality traits.

## Conclusions

Overall the data supported the idea that significant changes in quality-related metabolites occur not only according to the ripening process but also depending on the genetic background (Carli et al., 2011). Consequently, metabolic profiling and the association of metabolic profiles with variation at specific genomic regions may represent a useful tool to characterize traditional varieties with functional markers in order to establish new criteria for distinctiveness and protection (Vallverdú-Queralt et al., 2011). The reported analysis indicated the reliability of the described association; turning these information into markers efficient for selection or into candidates for cloning the genes underlying mQTLs will need the study of a much wider germplasm collection, endowed with wider phenotypic diversity.

In the past decade, the platforms for genotyping plant genomes at high density have increased considerably due to resequencing (Shirasawa et al., 2013; Ercolano et al., 2014; Lin et al., 2014) and genotyping by sequencing (Deschamps et al., 2012) approaches. In parallel, opportunities for efficiently analyzing a large number of genotypes for phenotypic as well as biochemical traits are becoming more affordable (Klee and Tieman, 2013). This scenario paves the way for investigating the genetic/molecular basis of organoleptic trait variation and breeding for quality-related compounds in tomato fruits. Network analysis demonstrated that the complex control of organoleptic quality in fresh tomato can be dissected into few strong relationships between sensory perception and specific biochemical data (Carli et al., 2009). This achievement supports the possibility of unraveling main genetic determinants of tomato quality and improving the crop by breeding a limited number of favorable alleles into elite germplasm.

## Author contributions

AM and VF designed the study. SB, MP, AP carried out the morphological characterization. AT and RF performed the biochemical analyses. VR, AB, and AM carried out the analysis of data and drafted the manuscript. All authors corrected and approved the final version.

## Funding

This work was supported by the Italian Ministry for Economic Development (MiSE), PROGRAMMA INDUSTRIA 2015, “Made in Italy”, TEMA A6, project title “Approcci TEcnologici Nuovi per l'Aumento della shelf-life e del contenuto di servizio nei prodotti qualificanti il modello alimentare mediterraneo” (ATENA). We are finally grateful to the COST Action FA1106 QualityFruit, supported by COST (European Cooperation in Science and Technology) for support to mobility.

### Conflict of interest statement

The authors declare that the research was conducted in the absence of any commercial or financial relationships that could be construed as a potential conflict of interest.
