# Supplementary material for: Metabolite Profiling of Italian Tomato Landraces with Different Fruit Types
Source: Front Plant Sci. 2016 May 19;7:664. doi: 10.3389/fpls.2016.00664 (PMC4872001; doi:10.3389/fpls.2016.00664)
Supplement: Supplementary file 2 [file Image1.PDF]

Supplementary Material

Metabolite Profiling of Italian Tomato Landraces with Different Fruit Types

Svetlana Baldina, Maurizio Enea Picarella, Antonio Dario Troise, Anna Pucci, Valentino Ruggieri, Rosalia Ferracane, Amalia Barone, Vincenzo Fogliano, Andrea Mazzucato*

*** Correspondence**: Andrea Mazzucato: mazz@unitus.it

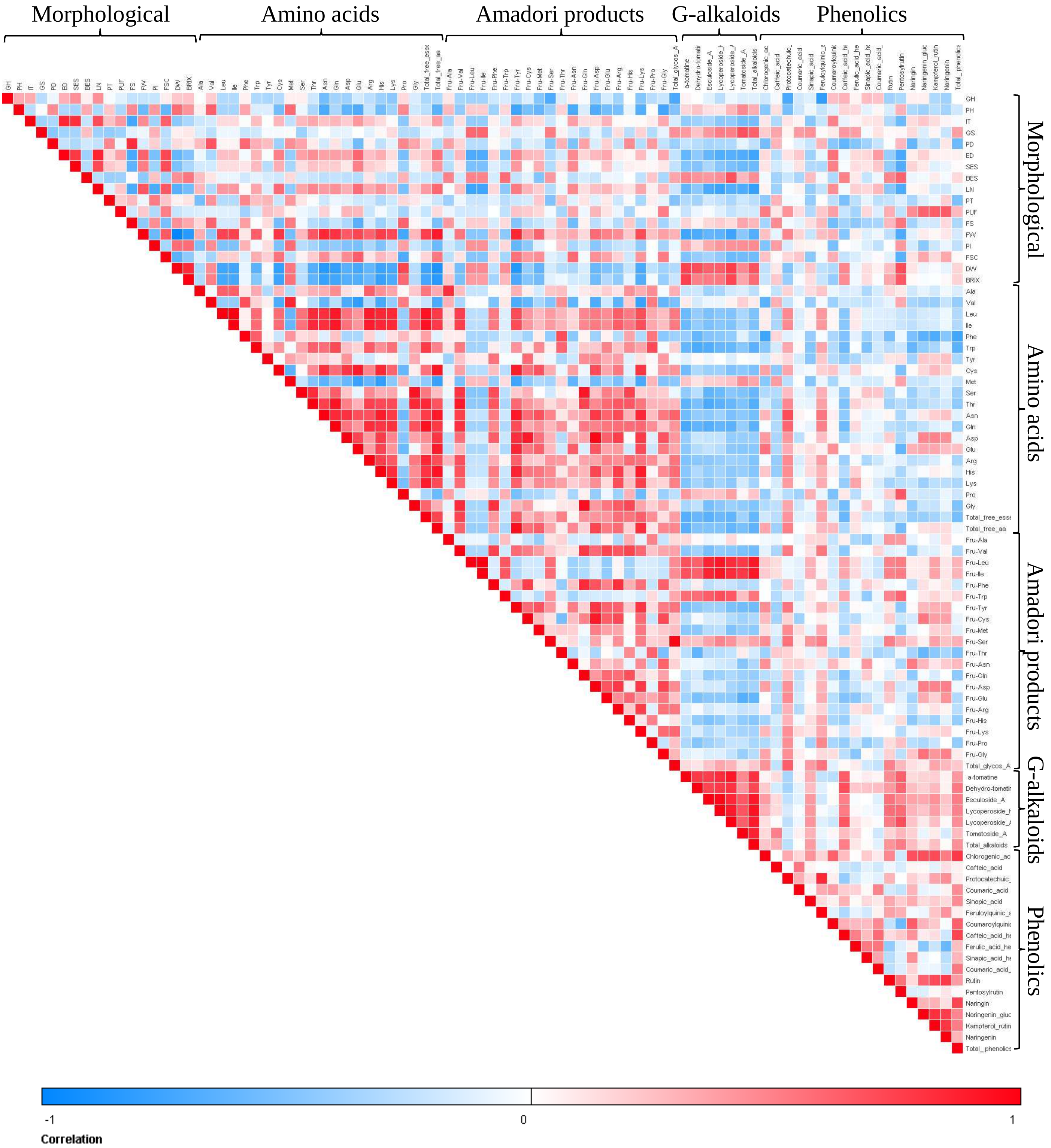

Supplementary Figure 1. Heatmap showing pair-wise correlations of 15 phenotypic, 2 proximate and 63 metabolic traits in the material analysed.

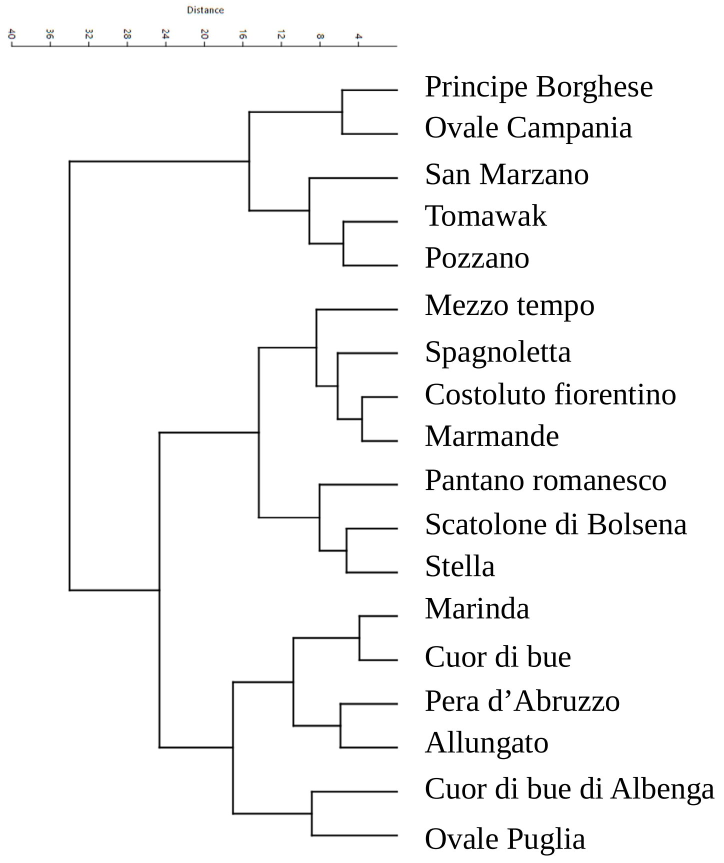

Supplementary Figure 2. Hierarchical clustering (Ward’s method) of the 15 landraces and three hybrids studied based on the content of 63 analyzed metabolites.

A

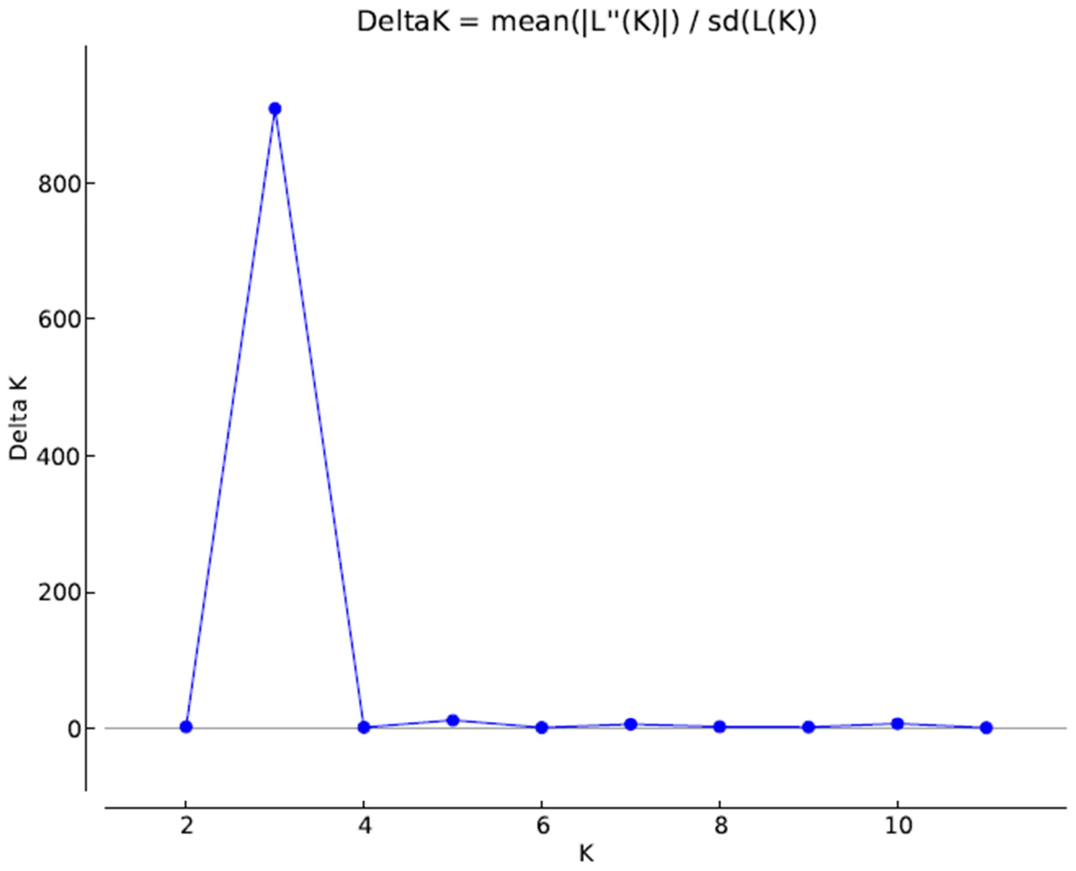

B

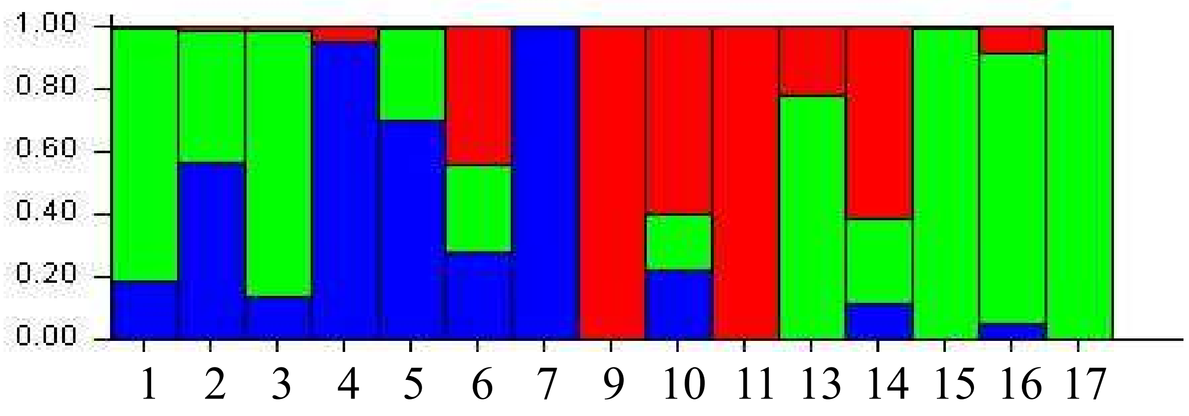

Supplementary Figure 3. Determination of Δ*K* using STRUCTURE. Δ*K* analysis for a different number of clusters (*K*) for the tomato accessions studied (A) and estimated population structure where each individual is represented by a vertical bar, which is partitioned into coloured segments that represent the individual estimated levels of the clusters (B).

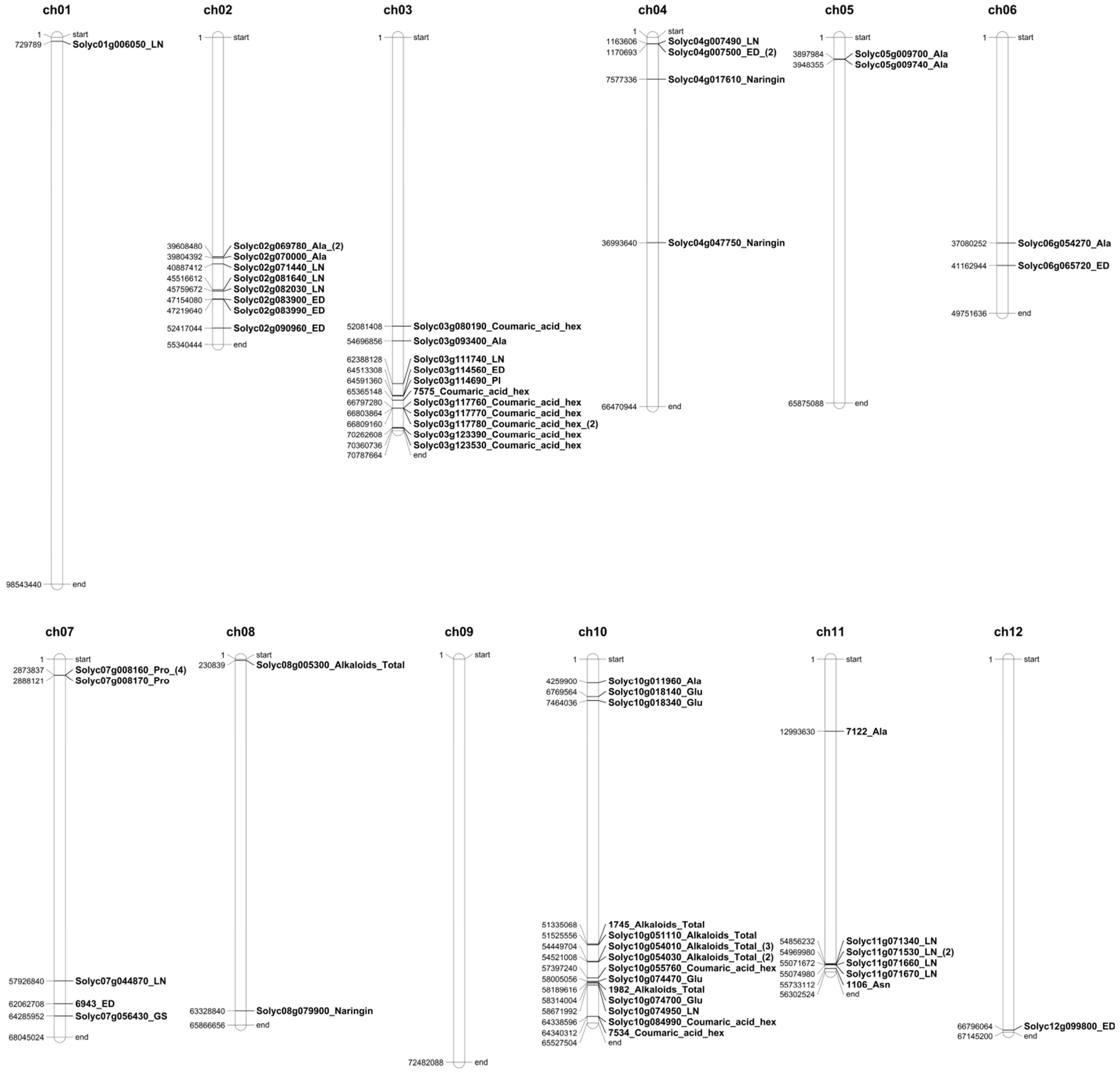

Supplementary Figure 4. Physical map of the tomato genome showing the position of the SNP markers associated to the studied morphological and metabolite traits. On the right of the chromosomes are reported the code of the gene harboring the SNP (or the SolCAP SNP code) and the associated trait. On the left the physical position according to SL2.50 is reported.
